# Retinal and Visual Pathways Involvement in Carriers of Friedreich’s Ataxia

**DOI:** 10.3390/diagnostics12123135

**Published:** 2022-12-12

**Authors:** Lucia Ziccardi, Lucilla Barbano, Giulio Antonelli, Ettore Cioffi, Antonio Di Renzo, Valeria Gioiosa, Christian Marcotulli, Andrzej Grzybowski, Carlo Casali, Vincenzo Parisi

**Affiliations:** 1 IRCCS—Fondazione Bietti, Via Livenza 1, 00198 Rome, Italy; 2 Department of Medical and Surgical Sciences and Biotechnologies, Sapienza University of Rome, 00185 Rome, Italy; 3 Department of Ophthalmology, University of Warmia and Mazury, Michała Oczapowskiego 2, 10455 Olsztyn, Poland; 4 Institute for Research in Ophthalmology, Foundation for Ophthalmology Development, Mickiewicza 24/3B, 60-836 Poznań, Poland

**Keywords:** Friedreich, ataxia, carriers, ERG, mfERG, VEP, PERG, OCT

## Abstract

Friedreich’s ataxia (FRDA) is a rare autosomal recessive neurodegenerative disorder due to the homozygous pathological expansion of guanine-adenine-adenine (GAA) triplet repeats in the first intron of the FXN gene, which encodes for the mitochondrial protein frataxin. In the visual system, the typical manifestations are ocular motility abnormality, optic neuropathy, and retinopathy. Despite the evidence of ophthalmological impairment in FRDA patients, there is a lack of information about the morpho-functional condition of the retina and of the optic pathways in healthy heterozygous carriers of Friedreich’s ataxia (C-FRDA). Ten C-FRDA subjects (providing 20 eyes) and thirty-five Controls (providing 70 eyes) underwent a complete neurological and ophthalmological examination comprehensive of functional (full-field Electroretinogram (ffERG), multifocal Electroretinogram (mfERG), Visual Evoked Potential (VEP), and Pattern Reversal Electroretinogram (PERG)) and morphological assessments (Optical Coherence Tomography, OCT) of the retina, macula, retinal ganglion cells, and visual pathways. The groups’ data were compared using a two-sample *t*-test. Pearson’s test was used to investigate the morpho-functional correlations. Statistically significant differences (*p* < 0.01) between C-FRDA and Control eyes for the values of the following parameters were found: ffERG b-wave amplitude, mfERG Response Amplitude Densities, PERG P50 implicit time and P50-N95 amplitude, VEP P100 implicit time, Retinal Nerve Fiber Layer (RNFL) Overall, and Nasal thickness. The values of the OCT macular volume were not statistically different (*p* > 0.01) between the two Groups. Therefore, our data suggest that, in C-FRDA, a dysfunction of retinal elements without morphological macular impairment may occur. In addition, a morphological impairment of RNFL associated with an abnormal neural conduction along the visual pathways can be also detected.

## 1. Introduction

Friedreich’s ataxia (FRDA), the most common inherited ataxia [1], is a rare autosomal recessive neurodegenerative disorder due to the homozygous pathological expansion of guanine-adenine-adenine (GAA) triplet repeats, commonly in the range of 70 to 1700, in the first intron of the FXN gene located on chromosome 9q13-q21.1, which encodes for the mitochondrial protein frataxin [2]. Few compound heterozygotes with expanded GAA repeats on one allele and a FXN point mutation on the other allele have also been described [1,2].

Frataxin is implicated in iron homeostasis [3] and hypersensitivity to oxidative stress [4]; therefore, its deficiency results in an abnormal influx of iron into the mitochondria, defective energy supply, generation of reactive oxygen species, and increasing the susceptibility of the nervous system, including the visual pathway, to oxidative stress [5]. Frataxin is found in high concentrations in the cells of the heart, spinal cord, liver, pancreas, and voluntary movements muscles [6]; thus, the reduced production induces specific multiorgan clinical characteristics, including neurological features such as ataxia of all four limbs, poor balance, impaired coordination, cerebellar dysarthria, weakness, ocular fixation instability, deep sensory loss, and visual and hearing impairment, and non-neurological involvement, such as hypertrophic cardiomyopathy, diabetes mellitus, kyphoscoliosis, and foot deformities, which are prototypical signs of FRDA.

Disease onset is often in the teens; however, late-onset (26–39 years) and very-late-onset (over 40 years)-affected FRDA have also been reported, usually presenting with a milder phenotype and lack of systemic manifestations [1]. The variability of clinical symptoms in FRDA disease, as well as the severity of the disease and the age of disease onset, are associated with the expansion size of GAA triplet repeats [1].

In overt FRDA disease, two-thirds of patients may display visual system impairment [7], ocular motor abnormalities being the most frequent and therefore well characterized in this neurological disorder [7]. Indeed, saccadic dysmetria, frequent square wave jerks [8], disruption of tracking movements, and impaired visual–vestibular interaction [9] have been reported, reflecting the disruption of the brainstem, cortical, and vestibular pathways. Other ophthalmological manifestations can be found in up to 30% of patients [7,10,11], including optic neuropathy and, less commonly, peripheral retinopathy [1,12] or pattern dystrophy [13].

In details, the morpho-function assessment of the visual pathways, as well as the optic nerve and the retinal ganglion cells (RGC), have been studied in affected FRDA by Visual Evoked Potentials (VEP), pattern Electroretinogram (PERG), and Optical Coherence Tomography (OCT). Optic nerve dysfunction detected by VEP associated with optic nerve pallor [11,14,15], as well as reduced RGC function observed by PERG, suggested a neurodegenerative process involving both optic nerve and inner retina in FRDA [14]. In addition, the morphological analysis by means of OCT of both the optic nerve and macular region showed a reduced thickness of the retinal nerve peripapillary fiber layers (RNFL) [7,12,16] of the RGC and of the macular layers, altogether considered signs of neurodegenerative processes [7,10,16,17].

Unaffected carriers of FRDA (C-FRDA) are defined as individuals lacking neurological signs and symptoms and therefore healthy, such as parents or siblings of patients with the typical form of FRDA, harboring in heterozygosity-expanded GAA repeats in the typical range between 600 and 1200 on one single allele [2].

Despite the evidence of full-blown ophthalmological impairment in affected FRDA, there is a lack of information about the morpho-functional condition of the retina and of the visual pathways in healthy heterozygous C-FRDA.

Thus, in the present study, after neurological and genetic assessment, we performed an extensive morpho-functional evaluation of the entire retina, the macula, the RGC, and of the visual pathways in C-FRDA. Our aim was to evaluate whether retinal and/or visual pathways’ functional and morphological abnormalities could be detectable in C-FRDA and to investigate on the potential correlations between retinal/macular and RGC involvement and the neural conduction along the visual pathways in the same subjects. Operatively, by performing an ophthalmological evaluation in sequentially referred patients affected by FRDA, we extended the full morpho-functional assessment of the retina and the visual pathways to their healthy family C-FRDA members to identify any functional or morphological abnormalities involving one or more structures of the visual system.

## 2. Materials and Methods

### 2.1. Study Design and Participants

This observational case series study has been performed in accordance with the statement of the Declaration of Helsinki at the Clinical and Research Center of Neurophthalmology, Genetic and Rare Diseases of IRCCS Fondazione Bietti in Rome on subjects referred by the Department of Medical and Surgical Science and Biotechnologies, Sapienza University of Rome, between October 2019 and March 2020. The study protocol was approved by the Institutional Review Board (Department of Medical and Surgical Science and Biotechnologies, Sapienza University of Rome) on 10 July 2019. Informed consent after full explanation of all procedures was obtained from each subject enrolled in the study.

Healthy family members of genetically confirmed FRDA patients, harboring GAA triplet repeats of the FXN gene on one allele (heterozygous state), named C-FRDA, were selected. Thirty-five healthy subjects served as Controls.

All participants (C-FRDA and Controls) underwent a complete neurological and ophthalmological examination (see below) comprehensive of functional and morphological assessments of the retina, the macula, the RGC, and the visual pathways.

Although few clinical studies have been conducted on C-FRDA subjects, limited extrapolated data report the absence of any symptoms but the susceptibility to develop diabetes [18]. Since it is known that diabetes may alter macular [19], RGC [20,21], and optic nerve function [21,22], we excluded all C-FRDA with a previous history of diabetes and with a HbA1c level > 6.5%.

After full neurological and ophthalmological examinations and exclusion of diabetic disease, 10 C-FRDA subjects (6 males and 4 females, mean age: 56.31 ± 6.40 - years ± standard deviation, SD) providing 20 eyes with Best Corrected Visual Acuity (BCVA) of 0.0 LogMAR (mean 0.0 ± 0.0) were enrolled in the study. They were all asymptomatic for neurological and ophthalmological signs and symptoms and heterozygotes with GAA triplet repetitions in the typical range between 600 and 1200 (mean 787.57 ± SD 127.91) (see Table S1).

Control healthy subjects were totally 35 (20 males and 15 females, mean age: 53.15 ± 4.51 years ± SD) providing 70 eyes with BCVA of 0.0 LogMAR (mean 0.0 ± 0.0).

### 2.2. Neurological and Non-Neurological Examination

All C-FRDA subjects included in the study were healthy carriers, parents or siblings of patients with typical form of FRDA. Clinical neurological manifestations of FRDA, such as cerebellar ataxia, movement disorders, pyramidal signs, peripheral nerve signs, dysarthria, and hearing impairment, as well as non-neurological involvement, such as cardiomyopathy, kyphoscoliosis, and foot deformities, were investigated. No neurological and non-neurological signs and symptoms were present in any C-FRDA. The HbA1c level, assessed by high-performance liquid chromatography, was < 6.5% in all C-FRDA.

### 2.3. Ophthalmological Evaluation

All participants underwent a full ophthalmological evaluation, including BCVA, intraocular pressure (IOP) measurement, color testing by monocular administration of Ishihara pseudoisochromatic plates (Kanehara Trading Inc. Tokyo, Japan), anterior segment observation by slit-lamp biomicroscopy, and fundus examination by indirect ophthalmoscopy (by +90 Volk lens) after pupil dilation (1% Tropicamide drops). All enrolled subjects were enclosed in the study based on the following inclusion criteria:

- absence of a mean refractive error > ±3.00 spherical equivalent;
- IOP less than 18 mmHg;
- absence of corneal or lens opacities;
- ability to maintain a stable fixation;
- absence of systemic diseases (i.e., blood hypertension and rheumatologic disorders)
- absence of intake of drugs (i.e., chloroquine and hydroxychloroquine) that may influence the retinal and optic nerve function or structure.

### 2.4. Visual Acuity Assessment

BCVA was evaluated by administering the modified self-illuminated Early Treatment Diabetic Retinopathy Study (ETDRS) charts (Lighthouse, Low Vision Products, Long Island, NY, USA) at the distance of 4 m. BCVA was measured as LogMAR.

### 2.5. Retinal and Macular Function Examination

The function of the entire retina was assessed by full-field Electroretinograms (ffERG). FfERGs were recorded according to the International Society for Clinical Electrophysiology of Vision (ISCEV) standards [23] by the Retimax system (CSO, Firenze, Italy). Each subject was adapted to the dark for 20 min. The pupils were maximally dilated with Tropicamide 1% eye drops. Local anesthesia was provided by the application of 0.4% Benoxinate eye drops. Active Dawson–Trick–Litzkow (DTL) lower eyelid contact electrodes and reference electrodes (Ag/AgCl skin electrode placed on the correspondent outer canthi) were used for binocular recordings. A small Ag/AgCl skin ground electrode was placed at the center of the forehead. Dark-adapted 0.01 ffERG protocol was used [23]. The signal was amplified (gain 5000), filtered (bandpass 0.3–300 Hz), and averaged with the automatic rejection of artifacts. The analysis time was 125 milliseconds (ms). The typical ffERG is a biphasic signal characterized by waves, two of which (the negative a-wave and the positive b-wave) have a mean implicit time of 16 and 40 ms, respectively, in normal subjects. We measured the amplitude of the b-wave (ffERG b-wave A) from the first negative trough and the first positive peak in microvolts (µV).

The function of the outer retina of the macular area was explored by a multifocal Electroretinogram (mfERG) by using the VERIS mfERG system (VERIS Clinic TM version 4.9; Electro-Diagnostic Imaging, San Mateo, CA, USA) following the updated 2021 ISCEV standards [24]. The multifocal stimulus, consisting of 61-scaled hexagons, was displayed on a high-resolution, black-and-white monitor (size, 32 cm width and 30 cm height) with a frame rate of 75 Hz. The array of hexagons subtended 20° of the visual field. Each hexagon was independently alternated between black (1 cd/m^2^) and white (200 cd/m^2^) according to a binary m-sequence. This resulted in a contrast of 99%. The luminance of the monitor screen and the central fixation cross (used as target) was 100 cd/m^2^. The m-sequence had 2^13−1^ elements, and the total recording time was approximately 4 min. The total recording time was divided into eight segments. Between segments, the subject was allowed to rest for a few seconds. Focusing lenses were used when necessary. To maintain a stable fixation, a small red target, which was perceived by all subjects tested, was placed in the center of the stimulation field. A camera provided an image of the eye, which was displayed on the computer screen so that fixation could be continuously monitored.

MfERGs were recorded in the presence of pupils that were maximally pharmacologically dilated with 1% Tropicamide eye drops to a diameter of 7–8 mm. The cornea was anaesthetized with 0.4% Benoxinate eye drops. The electrodes settings were the same used for the ffERG recordings. In the analysis of the mfERG responses, we considered, for each obtained averaged response, the peak-to-peak response amplitude density (RAD) measured in nanovolt/degree^2^ (nV/d^2^) between the first negative peak (N1) and the first positive peak (P1) of the waveforms.

As already published in our previous works [19,25], we explored the bioelectrical responses derived from concentric annular retinal areas (rings) centered on the fovea. The averaged responses were obtained from five rings with increasing eccentricity from the fovea: from 0 to 2.5 degrees (ring 1, R1), from 2.5 to 5 degrees (ring 2, R2), from 5 to 10 degrees (ring 3, R3), from 10 to 15 degrees (ring 4, R4), and from 15 to 20 degrees (ring 5, R5).

### 2.6. RGC and Visual Pathways Function Examination

Simultaneous Pattern Electroretinogram (PERG) and Visual Evoked Potential (VEP) recordings were performed following our previously published methods [26,27,28] to assess the RGC and the visual pathway’s function, respectively.

Subjects were seated in a semi-dark, acoustically isolated room in front of the display and surrounded by a uniform field of luminance of 5 cd·m^2^ for monocular recordings. We used a visual stimulus of a checkerboard pattern (contrast 80%, mean luminance 110 cd/m^2^) generated on a television monitor and reversed in contrast at the rate of 2 reversals per second. A small fixation target, subtending a visual angle of approximately 0.5 degrees (estimated after considering spectacle-corrected individual refractive errors) was placed at the center of the pattern stimulus. At the viewing distance of 114 cm, in the monitor screen subtending 23 degrees, the checked edges subtended 15 min (15ʹ) for the PERG recordings and 15ʹ and 60′ of the visual angle for the VEP recordings. Therefore, PERG was recorded in response to 15′ checks, whereas, for VEP, we used two different checkerboard patterns, as suggested by the ISCEV standards [29], to obtain a prevalent activation of smaller (15ʹ checks) and larger (60ʹ checks) axons of the optic pathways [26,30], respectively.

The setting for the 15′ PERG recording was done by a small Ag/AgCl skin electrode placed over the lower eyelid. PERG signals were derived between the stimulated (active electrode) and the patched (reference electrode) eyes using a previously described method [31]. The ground electrode was in Fpz. Interelectrode resistance was lower than 3000 ohms. The signal was amplified (gain 50.000), filtered (bandpass 1–30 Hz), and averaged with the automatic rejection of artefacts (100 events free from artefacts were averaged for every trial) by Retimax (CSO, Firenze, Italy). The analysis time was 250 ms. In the analysis of the PERG responses, we considered the 15′ implicit time (IT) of the P50 peak (PERG IT, measured in ms) and the 15′ peak-to-peak amplitude (A) between the P50 and the N95 peaks (PERG P50-N95 amplitude, PERG A, measured in microvolts).

The settings for the 15′ and 60′ VEP recordings made of cup-shaped electrodes of Ag/AgCl were fixed with conductive paste in the following positions: active electrode in Oz, reference electrode in Fpz, and ground in the left arm. The interelectrode resistance was kept below 3000 ohms. The bioelectric signal was amplified (gain 20.000), filtered (bandpass 1–100 Hz), and averaged (200 events free from artefacts were averaged for every trial) by Retimax (CSO, Firenze, Italy). The analysis time was 250 ms. We analyzed for the 15′ and 60′ recordings, the IT of the P100 peak (VEP IT, measured in ms) and the peak-to-peak A between the N75 and the P100 peaks, VEP N75-P100 amplitude (VEP A, measured in microvolts).

### 2.7. Macula and Optic Nerve Morphological Examination

The morphology of the macular area and of the RNFL of the optic nerve can be explored in vivo by OCT, providing layer-by-layer objective measurements of anatomical structures.

Macular volume (MV) and RNFL thickness (RNFL-T) were assessed using the RTVue-100 Sd-OCT device (RTVue Model-RT100 version 6.3; Optovue Inc., Fremont, CA, USA) according to APOSTEL’s recommendations [32].

OCT scans were obtained in a dark room after pupil dilation with Tropicamide 1% eye drops, and each scan was carefully reviewed for the accurate identification and segmentation of the retinal layers by two expert graders (LZ and LB) to exclude cases of failed segmentation. The OCT image quality signal strength index of the acquired scan was at least 40. Scans that did not fulfil the above criteria were excluded from the analysis. The RTVue-100 device uses a low coherence light source centered at 840 nm with a 50 nm bandwidth, which gives an axial resolution of 5 μm. By using the MM5 protocol, we collected MV data from the ETDRS map. The MM5 grid scanning protocol consists of 11 horizontal lines with a 5 mm scan length, 6 horizontal lines with a 3 mm scan length, 11 vertical lines with a 5 mm scan length, and 6 vertical lines with a 3 mm scan length each at 0.5 mm intervals, all centered at the fovea. The number of A-scans in long horizontal and vertical lines is 668, and the number of A-scans in short horizontal and vertical lines is 400. This scan configuration provided an acquisition rate of 26.000 A-scans/second. The segmentation algorithm of the MM5 scanning protocol also enables the automatic segmentation of the whole retinal volume (MV-WR), the inner retinal volume (MV-IR), and the outer retinal volume (MV-OR) of the macular region. The software automatically divides the inner and outer neurosensory retinas at the boundary between the inner nuclear layer (INL) and the outer plexiform layer (OPL). The IR examines the RNFL, the complex of RGC, and inner plexiform layer (GC/IPL) and the INL. The OR encloses the OPL, the outer nuclear layer (ONL), and the photoreceptor layer. The boundaries of the OR were the anterior of the OPL and the photoreceptor inner segment/outer segment junction. The following boundaries were identified for the IR segmentation: the inner limiting membrane and the posterior of the INL.

Peripapillary RNFL 3.45 protocol was used. The characteristics of the OCT evaluation are reported extensively in our previous work [33]. In the OCT results, we considered the average value of the RNFL thickness of the following quadrants: superior (RNFL-ST), inferior (RNFL-IT), nasal (RNFL-NT), and temporal (RNFL-TT); the overall thickness obtained in all quadrants (average of 8 values) was identified as RNFL overall (RNFL-OT).

### 2.8. Statistical Analysis

The sample size was calculated by using preliminary groups of 5 participants, respectively, for C-FRDA and Controls. The size evaluation is based on the following mfERG R1 RAD values: 65 ± 9 nV/deg^2^ for Controls and 45 ± 12 nV/deg^2^ for C-FRDA at α = 1% (type 1 error) and power = 90% (β = 10%), giving us 10 participants for each group.

The groups’ demographic data were compared using an unpaired two-sample *t*-test (age: *t* = 1.46, *p* = 0.173) and χ^2^ test (gender: χ^2^ = 0.026, *p* = 0.872). We compared electrophysiological data from the Control and C-FRDA groups using an unpaired two-sample *t*-test to evaluate whether the groups’ parameters would differ.

Descriptive statistics (Table 1) were shown as the mean and SD. Table 2 included inferential statistics (t, p). In all analyses, a more conservative *p*-value ≤ 0.01 was considered statistically significant.

Moreover, the relationships between macular functional data (mfERG RAD from R1, R2, and R3 and 15′ PERG IT and A) and morphological (MV-OR and MV-IR) were investigated in C-FRDA by means of a Pearson’s test (Table 3). Similarly (Table 4), the RNFL thickness of the temporal sector (RNFL-TT) was correlated with the RGC and papillomacular bundle function (15′ PERG and VEP IT and A), and the overall RNFL thickness (RNFL-OT) was correlated with the large axons neural conduction (60′ VEP IT and A). Further, the number of GAA triplet repetitions was correlated with all the retinal and optic nerve functional and morphological data (see Table S2). As stated before, a *p*-value ≤ 0.01 was considered statistically significant.

SPSS (version 25) software was used for the statistical analysis. IBM SPSS Statistics for Windows version 25 (IBM Corp, Armonk, NY, USA)

## 3. Results

An ophthalmological examination did not show any abnormalities in the fundus appearance in all C-FRDA examined eyes, with no evident macular, peripheral retina, blood vessels, or optic nerve changes.

**Figure 1:**
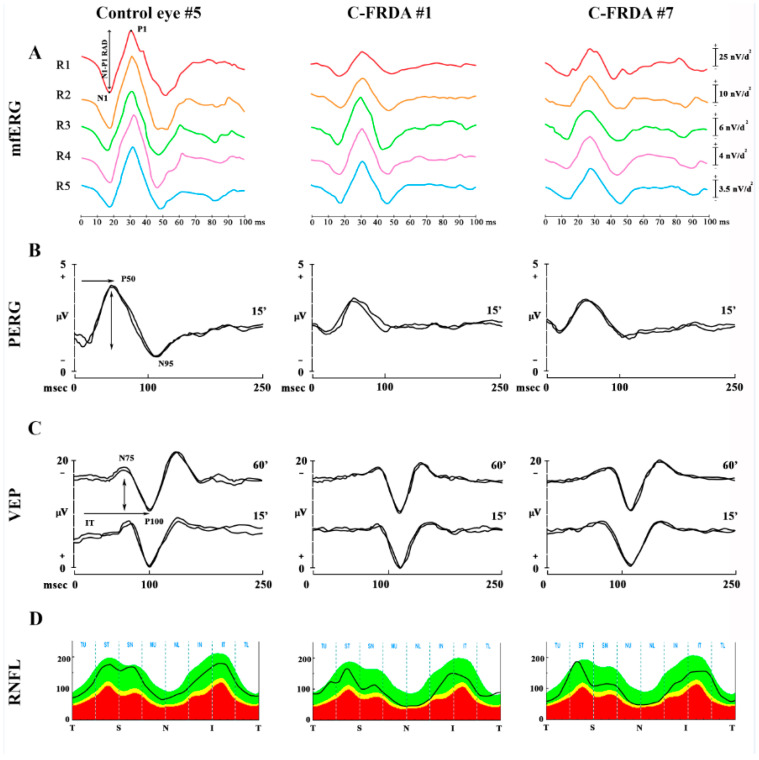
Examples of Multifocal Electroretinogram (mfERG) recordings (**A**), Pattern Electroretinogram (PERG) (**B**), Visual Evoked Potentials (VEP) (**C**), and Optical Coherence Tomography (OCT) Retinal Nerve Fiber Layer (RNFL) thickness (**D**) from the eyes of one representative control subject and two subject carriers for Friedreich’s ataxia (C-FRDA). (**A**) The mfERG Control eye traces the N1-P1 (↕) response amplitude density (RAD, measured in nanoVolt/degree^2^—nV/deg^2^); it is obtained from five concentric annular retinal regions (rings) centered on the fovea: from 0 to 2.5 degrees (ring 1, R1, in red), from 2.5 to 5 degrees (ring 2, R2, in orange), from 5 to 10 degrees (ring 3, R3, in green), from 10 to 15 degrees (ring 4, R4, in purple), and from 15 to 20 degrees (ring 5, R5, in blue). The RAD values are reduced in all rings in C-FRDA eyes with respect to the Control eyes. (**B**) In the PERG examples, 15′ refers to checked edges subtending 15 min (15′) of the visual angle for pattern reversal visual stimuli; P50 and N95 refer to the first positive and the second negative peak, whose P50 implicit time (IT, →) and P50-N95 peak-to-peak amplitude (A, ↕) were considered. A reduced 15′ PERG A and a delayed 15′ PERG IT are found in the C-FRDA eyes respect to the Control eyes. (**C**) In VEP examples, 60′ and 15′ refer to checked edges subtending 60 min (60′) and 15 min (15′) of the visual angle for pattern reversal visual stimuli, respectively; N75 and P100 refer to the first negative and the first positive peaks of VEP recordings, whose P100 IT (→) and N75-P100 peak-to-peak A (↕) were considered. 15′ VEP IT and 60′ VEP IT are delayed in C-FRDA eyes with respect to Control eyes. (**D**) In the OCT RNFL analysis, T refers to the temporal sector, S refers to the superior sector, N refers to the nasal sector, and I refers to the inferior sector. The average of the values detected in each sector is considered. The RNFL thickness values in the nasal sector are reduced in C-FRDA eyes with respect to the Control eyes.

In Figure 1 are reported representative examples of mfERG recordings (Figure 1A), PERG and VEP recordings (Figure 1B,C), and OCT RNFL (Figure 1D) assessment detected in one control subject and in two C-FRDA eyes.

In Table 1, the results of the statistical descriptive analysis of the morpho-functional values (mean and SD values) of the following parameters are presented: BCVA, ffERG b- wave A; mfERG RAD from R1, R2, R3, R4, and R5; 15′ PERG IT and A; 15′ and 60′ VEP IT and A; MV-WR, MV-IR, and MV-OR; and RNFL-OT, ST, IT, NT, and TT obtained from 20 C-FRDA eyes and from 70 Controls eyes.

**Table 1:** Descriptive statistics: mean and standard deviation values of the functional and morphological retina and optic nerve parameters in carriers of Friedreich’s ataxia (C-FRDA) eyes and Control eyes (C).

	C (Mean; SD ^a^) n° ^b^ 70	C- FRDA (Mean; SD ^a^) n° ^b^ 20
BCVA (LogMAR) ^c^	0.00; 0.00	0.00; 0.00
ffERG b-wave A ^d^ (μV) ^e^	207.17; 31.59	158.58; 31.15
mfERG ^f^ R1 ^g^ RAD ^h^ (nV/deg^2^) ^i^	67.43; 8.8	47.91; 11.70
mfERG ^f^ R2 ^l^ RAD ^h^ (nV/deg^2^) ^i^	29.43; 6.3	21.13; 5.01
mfERG ^f^ R3 ^m^ RAD ^h^ (nV/deg^2^) ^i^	17.44; 3.2	11.86; 3.14
mfERG ^f^ R4 ^n^ RAD ^h^ (nV/deg^2^) ^i^	10.52; 1.3	7.75; 1.96
mfERG ^f^ R5 ^o^ RAD ^h^ (nV/deg^2^) ^i^	7.99; 1.0	6.36; 1.61
15′ ^p^ PERG ^q^ IT ^r^ (ms) ^s^	50; 3	55.81; 3.8
15′ ^p^ PERG ^q^ A ^t^ (μV) ^e^	2.48; 0.18	1.82; 0.46
15′ ^p^ VEP ^u^ IT ^v^ (ms) ^s^	104.42; 3.86	113.90; 9.34
15’ ^p^ VEP ^u^ A ^w^ (μV) ^e^	10.62; 2.15	9.24; 3.95
60′ ^x^ VEP ^u^ IT ^v^ (ms) ^s^	102.37; 3.41	111.15; 8.32
60′ ^x^ VEP ^u^ A ^w^ (μV) ^e^	11.56; 1.87	10.96; 3.23
MV ^y^—WR ^z^ (mm^3^) ^aa^	7.48; 0.31	6.40; 1.73
MV ^y^—IR ^ab^ (mm^3^) ^aa^	2.97; 0.21	2.76; 0.33
MV ^y^—OR ^ac^ (mm^3^) ^aa^	4.43; 0.37	4.46; 0.38
RNFL ^ad^ OT ^ae^ (μm) ^af^	113.90; 4.61	104.92; 9.91
RNFL ^ad^ ST ^ag^ (μm) ^af^	132.30; 11.24	126.27; 17.06
RNFL ^ad^ IT ^ah^ (μm) ^af^	140.50; 10.86	133.68; 20.44
RNFL ^ad^ NT ^ai^ (μm) ^af^	96.40; 9.15	77.31; 13.78
RNFL ^ad^ TT ^al^ (μm) ^af^	86.40; 8.62	81.26; 7.91

^a^ SD = standard deviation; ^b^ n° = number of enrolled eyes; ^c^ BCVA = Best correct visual acuity measured in LogMAR; ^d^ ffERG b wave A = amplitude of b wave obtained by full field electroretinogram recording; ^e^ μV = microvolt; ^f^ mfERG = multifocal electroretinogram; ^g^ R1 = ring 1 (circular retinal area centered on the fovea: from 0 to 2.5 degrees); ^h^ RAD = response amplitude density; ^i^ nV/deg^2^ = nanoVolt/degrees^2^; ^l^ R2 = ring 2 (concentric annular retinal area centered on the fovea: from 2.5 to 5 degrees); ^m^ R3 = ring 3 (concentric annular retinal area centered on the fovea: from 5 to 10 degrees); ^n^ R4 = ring 4 (concentric annular retinal area centered on the fovea: from 10 to 15 degrees); ^o^ R5 = ring 5 (concentric annular retinal area centered on the fovea: from 15 to 20 degrees); ^p^ 15′ = responses obtained with checks edges subtending 15 min of arc; ^q^ PERG = pattern electroretinogram; ^r^ IT = P50 implicit time; ^s^ ms = milliseconds; ^t^ A: P50-N95 amplitude; ^u^ VEP = visual evoked potential; ^v^ IT = P100 implicit time; ^w^ A: N75-P100 amplitude; ^x^ 60′ = responses obtained with checks edges subtending 60 min of arc; ^y^ MV = macular volume; ^z^ WR = whole retina; ^aa^ mm^3^ = cubic millimeters; ^ab^ IR = inner retina; ^ac^ OR = outer retina; ^ad^ RNFL = retinal nerve fibers layer; ^ae^ OT = overall thickness; ^af^ μm = micron; ^ag^ ST = superior thickness; ^ah^ IT = inferior thickness; ^ai^ NT = nasal thickness; ^al^ TT = temporal thickness.

In Table 2, the results of the inferential statistics are reported. On average, statistically significant differences (*p* < 0.01) between C-FRDA and Control Groups were found for the following values: ffERG b-wave A; mfERG RADs from R1, R2, R3, R4, and R5; 15′ PERG IT and A; 15′ and 60′ VEP IT; RNFL-OT; and RNFL-NT. By contrast, the mean values of BCVA; 15′ and 60′ VEP A; MV-WR, MV-IR, and MV-OR; and RNFL-ST, IT, and TT were not significantly different (*p* > 0.01) between the C-FRDA and Controls. In Figure S1 are presented the box plots of mfERG RAD values detected in the Controls and in the C-FRDA groups obtained from five concentric annular retinal regions (RINGS) centered on the fovea.

**Table 2:** Inferential statistics of the functional and morphological retinal and optic nerve values between carriers of Friedreich’s ataxia (C-FRDA) eyes and Control eyes (C).

	C vs. C-FRDA (t ^a^; p ^b^)
BCVA (LogMAR) ^c^	0.00; 1.00
ffERG b-wave A ^d^ (μV) ^e^	*5.12; <0.001*
mfERG ^f^ R1 ^g^ RAD ^h^ (nV/deg^2^) ^i^	*4.66; <0.001*
mfERG ^f^ R2 ^l^ RAD ^h^ (nV/deg^2^) ^i^	*3.46; 0.003*
mfERG ^f^ R3 ^m^ RAD ^h^ (nV/deg^2^) ^i^	*4.24; <0.001*
mfERG ^f^ R4 ^n^ RAD ^h^ (nV/deg^2^) ^i^	*4.16; <0.001*
mfERG ^f^ R5 ^o^ RAD ^h^ (nV/deg^2^) ^i^	*3.05; 0.006*
15′ ^p^ PERG ^q^ IT ^r^ (ms) ^s^	*−5.14; <0.001*
15′ ^p^ PERG ^q^ A ^t^ (μV) ^e^	*5.47; <0.001*
15′ ^p^ VEP ^u^ IT ^v^ (ms) ^s^	*−4.25; <0.001*
15’ ^p^ VEP ^u^ A ^w^ (μV) ^e^	1.40; 0.173
60′ ^x^ VEP ^u^ IT ^v^ (ms) ^s^	*−4.42; <0.001*
60′ ^x^ VEP ^u^ A ^w^ (μV) ^e^	0.78; 0.440
MV ^y^—WR ^z^ (mm3) ^aa^	2.62; 0.036
MV ^y^—IR ^ab^ (mm3) ^aa^	2.03; 0.053
MV ^y^—OR ^ac^ (mm3) ^aa^	−0.57; 0.573
RNFL ^ad^ OT ^ae^ (μm) ^af^	*3.94; 0.001*
RNFL ^ad^ ST ^ag^ (μm) ^af^	2.13; 0.043
RNFL ^ad^ IT ^ah^ (μm) ^af^	1.64; 0.114
RNFL ^ad^ NT ^ai^ (μm) ^af^	*5.12; <0.001*
RNFL ^ad^ TT ^al^ (μm) ^af^	1.60; 0.117

^a^ t = Student’s *t*-test; ^b^ p = *p*-value (less or equal (≤) to 0.01 was considered statistically significant); ^c^ BCVA = Best correct visual acuity measured in LogMAR; ^d^ ffERG b-wave A = amplitude of b-wave obtained by full field electroretinogram; ^e^ μV = microvolt; ^f^ mfERG = multifocal electroretinogram; ^g^ R1 = ring 1 (circular retinal area centered on the fovea: from 0 to 2.5 degrees); ^h^ RAD = response amplitude density; ^i^ nV/deg^2^ = nanoVolt/degrees^2^; ^l^ R2 = ring 2 (concentric annular retinal area centered on the fovea: from 2.5 to 5 degrees); ^m^ R3 = ring 3 (concentric annular retinal area centered on the fovea: from 5 to 10 degrees); ^n^ R4 = ring 4 (concentric annular retinal area centered on the fovea: from 10 to 15 degrees); ^o^ R5 = ring 5 (concentric annular retinal area centered on the fovea: from 15 to 20 degrees); ^p^ 15′ = responses obtained with checks edges subtending 15 min of arc; ^q^ PERG = pattern electroretinogram; ^r^ IT = P50 implicit time; ^s^ ms = milliseconds; ^t^ A: P50-N95 amplitude; ^u^ VEP = visual evoked potential; ^v^ IT = P100 implicit time; ^w^ A: N75-P100 amplitude; ^x^ 60′ = responses obtained with checks edges subtending 60 min of arc; ^y^ MV = macular volume; ^z^ WR = whole retina; ^aa^ mm^3^ = cubic millimeters; ^ab^ IR = inner retina; ^ac^ OR = outer retina; ^ad^ RNFL = retinal nerve fibers layer; ^ae^ OT = overall thickness; ^af^ μm = micron; ^ag^ ST = superior thickness; ^ah^ IT = inferior thickness; ^ai^ NT = nasal thickness; ^al^ TT = temporal thickness. On *italic* are reported the abnormal values.

As shown in Table 3, no significant (*p* > 0.01) linear correlations were found between the values describing the function of the outer and inner macular layers (mfERG RAD from R1, R2, and R3 and 15′ PERG IT and A) and the values describing the morphological conditions of the outer and inner macular layers (MV-OR and MV-IR) in C-FRDA eyes.

**Table 3:** Correlation between the morphological and functional values of the outer and inner macular region in carriers of Friedreich’s ataxia (C-FRDA).

	mfERG ^a^ R1 ^b^ RAD ^c^ (r ^f^; p ^g^)	mfERG ^a^ R2 ^d^ RAD ^c^ (r ^f^; p ^g^)	mfERG ^a^ R3 ^e^ RAD ^c^ (r ^f^; p ^g^)
MV ^h^-OR ^j^	0.39; 0.165	0.57; 0.031	0.54; 0.039
	**15′ ^j^ PERG ^k^ IT ^l^** **(r ^f^; p ^g^)**	**15′ ^j^ PERG ^k^ A ^m^** **(r ^f^; p ^g^)**	
MV ^h^–IR ^n^	−0.06; 0.835	0.23; 0.393	

^a^ mfERG = multifocal electroretinogram; ^b^ = R1 = ring 1 (circular retinal area centered on the fovea: from 0 to 2.5 degrees); ^c^ RAD = response amplitude density measured in nanoVolt/degrees^2^; ^d^ = R2 = ring 2 (concentric annular retinal area centered on the fovea: from 2.5 to 5 degrees); ^e^ R3 = ring 3 (concentric annular retinal area centered on the fovea: from 5 to 10 degrees); ^f^ r = Pearson’s correlation coefficient; ^g^ p = *p*-value of correlation (less or equal (≤) to 0.01 was considered statistically significant); ^h^ MV = macular volume measured in cubic millimeters; ^i^ OR = outer retina; ^j^ 15′ = responses obtained with checks edges subtending 15 min of arc; ^k^ PERG = pattern electroretinogram; ^l^ IT = P50 implicit time; ^s^ ms = milliseconds; ^m^ A: P50-N95 amplitude; ^n^ IR = inner retina.

As shown in Table 4, when we correlated the morphological values of the temporal sector of the optic nerve (RNFL-TT) with the functional values describing the neural conduction along the papillomacular bundle (15′ PERG and VEP IT and A), we found a statistically significant (*p* < 0.01) correlation between RNFL-TT and 15′ VEP IT. By contrast, no statistically significant (*p* > 0.01) morpho-functional correlations were found when we correlated the overall thickness of RNFL (RNFL-OT) with the functional values describing the neural conduction along the larger axons of the optic nerve (60′ VEP IT and A).

**Table 4:** Correlation between the morphological and functional values of retinal ganglion cells and optic nerve in carriers of Friedreich’s ataxia (C-FRDA).

	15′ ^a^ PERG ^b^ IT ^c^ (r ^d^; p ^e^)	15′ ^a^ PERG ^b^ A ^f^ (r ^d^; p ^e^)	15′ ^a^ VEP ^g^ IT ^h^ (r ^d^; p ^e^)	15’ ^a^ VEP ^g^ A ^i^ (r ^d^; p ^e^)
**RNFL TT ^j^**	−0.02; 0.926	0.13; 0.625	*−0.76; 0.001*	−0.02; 0.943
	**60′ ^k^ VEP ^g^ IT ^h^ (r ^d^; p ^e^)**	**60′ VEP ^g^ A ^i^ (r ^d^; p ^e^)**		
**RNFL OT ^l^**	−0.06; 0.798	0.26; 0.262		

^a^ 15′ = responses obtained with checks edges subtending 15 min of arc; ^b^ PERG = pattern electroretinogram; ^c^ IT = P50 implicit time measured in milliseconds; ^d^ r = Pearson’s correlation coefficient; ^e^ p = *p*-value of correlation (less or equal (≤) to 0.01 was considered statistically significant); ^f^ A: P50-N95 amplitude measured in microvolt; ^g^ VEP = Visual Evoked Potential; ^h^ IT = P100 implicit time measured in milliseconds; ^i^ A: N75-P100 amplitude measured in microvolt; ^j^ RNFL TT = retinal nerve fibers layer of temporal thickness measured in micron; ^k^ 60′ = responses obtained with checks edges subtending 60 min of arc; ^l^ RNFL OT = retinal nerve fibers layer of overall thickness measured in micron. In italics are reported the abnormal values.

When we correlated the functional mean values describing the RGC function (15′PERG A) with the mean values describing the neural conduction along the smaller axons of the visual pathways (15′VEP IT), no statistically significant (*p* = 0.187) correlations were found.

Moreover, when we correlated the functional and morphological values of the retina and optic nerves with the number of GAA triplet repetition, we observed a statistically significant correlation (*p* < 0.01) only between the number of triplet repetitions and ffERG b-wave A (See Table S2). The correlation between the number of triplet repetitions and the other morpho-functional parameters did not show any statistically significant results.

## 4. Discussion

In this observational study, we investigated, for the first time, the function and the morphology of the retina and of visual pathways in healthy carriers of FRDA. Since it is known that patients with overt FRDA may display abnormal RGC function (expressed by reduced PERG amplitude) and optic nerve abnormalities up to the level of optic atrophy with a delay of neuronal conduction along the large and the small axons of the optic pathways (detected by increased 60′ and 15′ VEP IT) [11,14], we were interested in evaluating whether retinal and/or visual pathways’ functional and morphological abnormalities could also be detectable in C-FRDA.

Therefore, we performed an extensive neurologic, genetic, and ophthalmological evaluation in C-FRDA to investigate the potential functional and morphological abnormalities of the entire retina, the macula, and the RGC and of the visual pathways and identify potential correlations between retinal/macular and RGC involvement and the neural conduction along the visual pathways in the same subjects. We excluded C-FRDA with a history of diabetes and HbA1c levels indicative of a diabetic status for the possible interference of diabetes on the function of the outer and inner retina and of the optic nerve [18,19,20,21,22].

### 4.1. Functional Abnormalities of the Retinal Preganglionic and Ganglionic Elements in C-FRDA

In the present study, we investigated the function of the entire retina by recording ffERG in C-FRDA subjects compared to the Controls. Our results of significantly reduced mean values of ffERG b-wave amplitude in C-FRDA eyes with respect to the Controls indicate a dysfunction of the entire outer retinal elements (mainly rod photoreceptors) in C-FRDA eyes (Table 2).

Moreover, we investigated the function of the preganglionic elements from localized macular areas, covering up to 20 degrees of eccentricity from the fovea, by recording mfERG and applying the ring analysis. When C-FRDA mfERG values were compared to those of the Controls, in C-FRDA, a significant reduction of mfERG RAD values in all examined rings (from R1 to R5) was found, and this might suggest an abnormal function of photoreceptors and bipolar cells located in the macular area (Table 2).

We also investigated the function of RGC, located in the innermost macular layers, by comparing 15′ PERG IT and A values between the Groups. In C-FRDA eyes, we observed significantly reduced 15′ PERG values with respect to the Controls (Table 2), indicating a dysfunction of RGC in the most central portion of the retina.

On macular morphology, by segmenting retinal layers though OCT, we did not find any significant differences between C-FRDA and Control eyes in the values of the whole retina and outer and inner macular volumes (Table 2), thus suggesting that the macular structures in our studied C-FRDA were not involved.

Additionally, when we correlated (see Table 3) the values describing the function of the preganglionic elements of the outer macular areas (mfERG RAD from R1, R2, and R3) with the values of the OR macular volume (MV-OR), on average, we did not find any significant functional–structural correlation in C-FRDA. These findings may suggest that a functional macular impairment that occurs in a condition of still preserved macular morphology could be detectable. Similarly, we correlated (see Table 3) the values describing the macular RGC function (15′ PERG IT and A) with the values of the IR macular volume (MV-IR), and no significant correlations were found in C-FRDA as well.

Moreover, we observed a significant correlation between the number of GAA triplets repetitions and the ffERG b-wave A. These data suggest that the dysfunction of the global retina occurs predominantly when the GAA triplets are expanded.

Therefore, all that described suggests that, in carriers of FRDA, there exists a functional retinal and macular impairment that is not related to structural macular abnormalities. It might be supposed that the preganglionic and the ganglionic elements dysfunction observed in the present study may be considered a manifestation of a neurodegenerative process involving retinal neuronal elements before the retinal layers display any structural impairment (as seen by the OCT and normal fundus appearance). In addition, this dysfunctional phenomenon does not impair visual acuity, since BCVA in C-FRDA eyes was similar to the Controls (Table 1).

From previous reports on different neurodegenerative diseases, such as diabetes type 1 [19] and Bardet-Biedl syndrome [34], it is known that retinal and macular dysfunction can be present in asymptomatic patients without clinical signs of retinal impairment. It might be the case that, in C-FRDA eyes, similar to other degenerative diseases, there exists a preclinical outer and inner retina/macular dysfunction.

As for the pre-ganglionic elements macular dysfunction observed in C-FRDA, in an animal mouse model of FRDA, signs of outer retinal degeneration with retinal pigmented epithelium thinning and photoreceptor loss have been reported, suggesting that low levels of frataxin correspond to an insufficient antioxidant capability to sustain photoreceptor vitality [35].

On the RGC, localized in the innermost layer of the macula, these are mostly involved in neurodegenerative processes involving brain disease by apoptotic phenomena [36]. It has been suggested that, in FDRA patients, reduced frataxin expression leads to decreased intracytoplasmic iron levels and shifts the redox state out of its optimum levels, predisposing the RGC toward oxidative damage. The progressive damage of RGC may gradually take place, and optic neuropathy would develop [37]. In our study, as suggested by the 15′ PERG IT and A abnormalities in absence from the MV-IR reduction and of a morpho-functional correlation, C-FRDA eyes showed a RGC dysfunction without inner retina morphological and ophthalmoscopic changes.

### 4.2. Functional and Morphological Abnormalities of the Neural Conduction along the Visual Pathways and of the Optic Nerve in C-FRDA

Our interest was also to identify any potential abnormalities on the optic nerve and of the neural conduction along the visual pathways in C-FRDA. We observed significantly increased 15′ and 60′ VEP IT mean values with respect to Control eyes (Table 2), thus describing significantly abnormal neuronal conduction along the small and the large axons of the visual pathways. Additionally, significantly reduced RNFL thickness overall and in the nasal sector was detected by OCT in C-FRDA.

In addition, when we correlated the morphological values of the temporal sector of the optic nerve (RNFL-TT) with the functional values describing the neural conduction along the small axons forming the papillomacular bundle (15′ VEP IT), we found a statistically significant correlation (Table 4), thus indicating a major morpho-functional involvement of the small axons of the optic nerve in carriers of FRDA. The abnormal neural conduction of the papillomacular bundle was not dependent on the RGC dysfunction (see the lack of correlation between 15′ PERG A and 15′ VEP IT), thus meaning that the functional retinal impairment and the abnormal neural conduction along the small axons of the optic nerve are independent in C-FRDA.

Taken together, our observations of impaired neural conduction along the large axons (delayed 60′ VEP IT) and of a linear correlation between the morpho-functional parameters of the papillomacular bundle (correlation between 15′ VEP IT with RNFL-TT), without the reduction of RNFL-TT, may represent novel findings in C-FRDA. These new insights might lead us to suppose that, in C-FRDA, there exists a dysfunction along both large and small axons of the visual pathways without the morphological involvement of the temporal sector of RNFT (normal RNFL-TT) and without a loss of visual acuity.

Moreover, our morphological findings on C-FRDA eyes showing reduced RNFL thickness overall and in the nasal sector with respect to Controls, suggesting a preferential structural impairment of those neuronal cells that project to the magnocellular pathways localized outside the macula and that do not specifically contribute to visual acuity [38]. These findings, together with the structural integrity of the outer macular layers seen by normal MV-OR values (see above), reinforce the reason why visual acuity is preserved in C-FRDA.

At the present, there is a lack of similar studies on C-FRDA, and our novel findings cannot be supported or contrasted by the available literature. Our ophthalmological data can be considered only in light of everything previously reported in FDRA disease referring exclusively to affected patients with or without visual symptoms (see Introduction [7,9,10,11,12,13,14,15,16,17,39].

In particular, our findings regarding the study of macular function by means of mfERG cannot be considered in relation to any previous study on FRDA, since, for the presence of motor ocular abnormalities or optic atrophy that limits the stable fixation required for the mfERG, this electrophysiological test was never carried out.

## 5. Conclusions

In conclusion, our data suggest that, in C-FRDA, a dysfunction of preganglionic and ganglionic retinal elements without morphological outer and inner retinal macular impairment may occur. In addition, a morphological impairment of RNFL associated with an abnormal neural conduction along the small and large axons of the optic nerve can be also detected.

All these observations, describing retinal and visual pathways impairment in C-FRDA, need to be confirmed by clinical studies with larger numbers of subjects, as well as by research investigations clarifying the role of frataxin in the visual system.

## Data Availability

Data are available upon request.

## Supplementary Materials

The following are available online at https://www.mdpi.com/article/10.3390/diagnostics12123135/s1, Figure S1: Box plots of Multifocal Electroretinogram (mfERG) response amplitude density (RAD, measured in nanoVolt/degree^2^—nV/deg^2^) values detected in Controls and in carriers for Friedreich Ataxia (C-FRDA) obtained from five concentric annular retinal regions (RINGS) centred on the fovea, Table S1: Individual data of carrier of Friedreich Ataxia (C-FRDA), Table S2: Correlations between functional and morphological retinal and optic nerve values with number of GAA triplet repetitions.
