# Supplementary material for: Retinal and Visual Pathways Involvement in Carriers of Friedreich’s Ataxia
Source: Diagnostics (Basel). 2022 Dec 12;12(12):3135. doi: 10.3390/diagnostics12123135 (PMC9777201; doi:10.3390/diagnostics12123135)

Table S1. Individual data of carrier of Friedreich Ataxia (C-FRDA).

Subject N°[a]	Age (years)	Genger (M[b]/F[c])	Number of GAA triplet ripetitions
C-FRDA #1	48	M[b]	822
C-FRDA #2	60	F[c]	843
C-FRDA #3	57	F[c]	1005
C-FRDA #4	48	F[c]	670
C-FRDA #5	51	F[c]	615
C-FRDA #6	65	M[b]	876
C-FRDA #7	65	M[b]	900
C-FRDA #8	58	M[b]	730
C-FRDA #9	55	M[b]	790
C-FRDA #10	53	M[b]	619

[a]N°= number of subject; [b]M = male; [c]F = female.

Table S2. Correlations between functional and morphological retinal and optic nerve values with number of GAA triplet repetitions.

	**Number of GAA triplet repetitions (r[a]; p[b])**
ffERG b-wave A[c] (μV)[d]	*-0.651; 0.003*
mfERG[e] R1[f] RAD[g] (nV/deg^2)[h]	-0.082; 0.779
mfERG[e] R2[i] RAD[g] (nV/deg^2)[h]	0.047; 0.873
mfERG[e] R3[j] RAD[g] (nV/deg^2)[h]	-0.215; 0.480
mfERG[e] R4[k] RAD[g] (nV/deg^2)[h]	-0.082; 0.781
mfERG[e] R5[l] RAD[g] (nV/deg^2)[h]	-0.182; 0.534
15′[m] PERG[n] IT[o] (msec)[p]	-0.105; 0.700
15′[m] PERG[n] A[q] (μV)[d]	-0.505; 0.046
15′[m] VEP[r] IT[o] (msec)[p]	-0.252; 0.285
15'[m] VEP[r] A[q] (μV)[d]	0.120; 0.615
60′[r] VEP[s] IT[t] (msec)[p]	-0.238; 0.312
60′[r] VEP[s] A[w] (μV)[d]	-0.003; 0.992
MV[x] – WR[y] (mm3)[z]	0.372; 0.106
MV[x] – IR[aa] (mm3)[z]	-0.018; 0.940
MV[x] – OR[ab] (mm3)[z]	-0.086; 0.719
RNFL[ac] OT[ad] (μm)[ae]	-0.156; 0.512
RNFL[ac] ST[af] (μm)[ae]	-0.397; 0.083
RNFL[ac] IT[ag] (μm)[ae]	0.069; 0.774
RNFL[ac] NT[ah] (μm)[ae]	-0.210; 0.374
RNFL[ac] TT[ai] (μm)[ae]	0.225; 0.341

[a]r= Pearson correlation coefficient ; [b]p= p-value of correlation (less or equal (⩽) to 0.01 was considered statistically significant); [c]ffERG b-wave A= amplitude of b-wave obtained by full field electroretinogram; [d]μV= microvolt; [e]mfERG = multifocal electroretinogram; [f]R1= ring 1 (circular retinal area centred on the fovea: from 0 to 2.5 degrees); [g]RAD=response amplitude density; [h]nV/deg^2= nanoVolt/$degrees^2$; [i]R2=ring 2 (concentric annular retinal area centred on the fovea: from 2.5 to 5 degrees); [j]R3= ring 3 (concentric annular retinal area centred on the fovea: from 5 to 10 degrees); [k]R4=ring 4 (concentric annular retinal area centred on the fovea: from 10 to 15 degrees); [l]R5=ring 5 (concentric annular retinal area centred on the fovea: from 15 to 20 degrees); [m]15′=responses obtained with checks edges subtending 15 minutes of arc; [n] PERG= pattern electroretinogram; [o]IT= P50 implicit time; [p] msec= milliseconds; [q]A: P50-N95 amplitude;

[r]60′=responses obtained with checks edges subtending 60 minutes of arc; [s]VEP= visual evoked potential; [t]IT= P100 implicit time; [w]A: N75-P100 amplitude; [x]MV= macular volume; [y]WR= whole retina; [z] mm3= cubic millimeters; [aa] IR= inner retina; [ab] OR= outer retina; [ac] RNFL= retinal nerve fibers layer; [ad] OT= overall thickness; [ae] μm= micron; [af] ST= superior thickness; [ag] IT= inferior thickness; [ah] NT= nasal thickness; [ai] TT= temporal thickness .

Figure S1. Box plots of Multifocal Electroretinogram (mfERG) response amplitude density (RAD, measured in nanoVolt/degree^2—nV/deg^2) values detected in Controls and in carriers for Friedreich Ataxia (C-FRDA) obtained from five concentric annular retinal regions (RINGS) centred on the fovea.

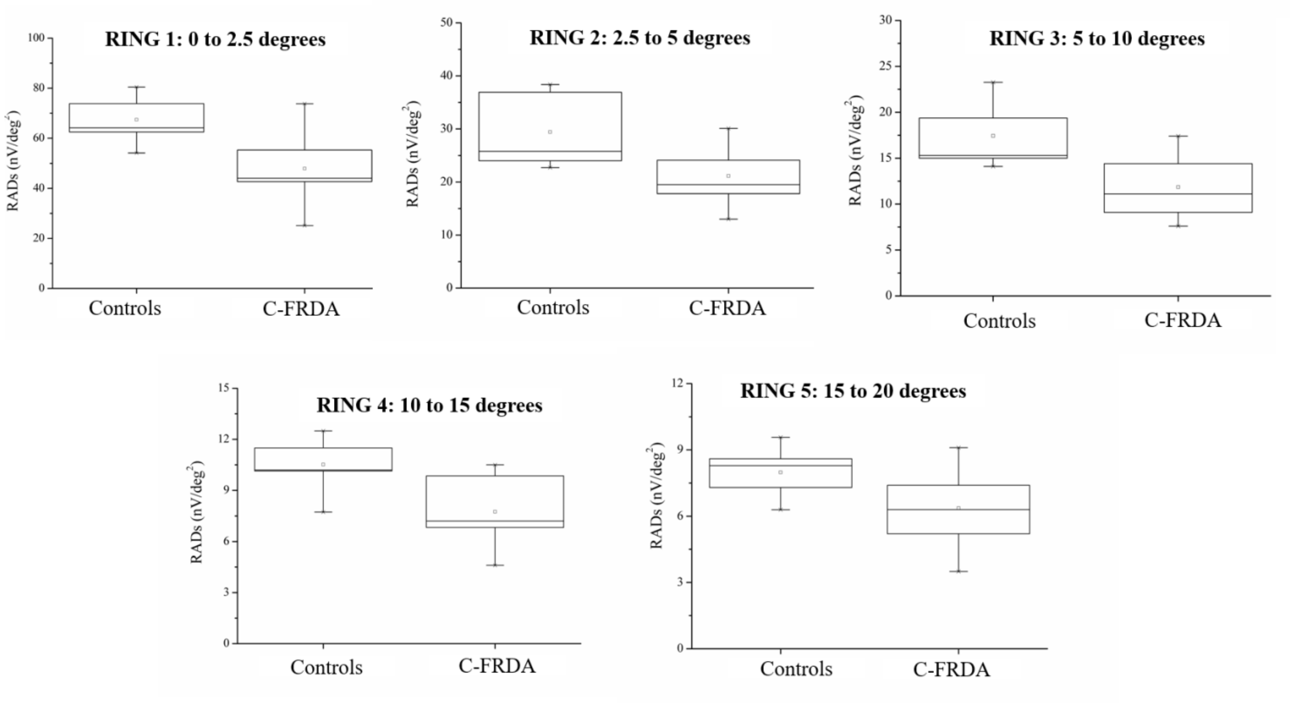

Supplement: Supplementary file 1 [file diagnostics-12-03135-s001.zip › diagnostics-2060744-supplementary.pdf]
